# ^68^Ga-PSMA and ^11^C-Choline comparison using a tri-modality PET/CT-MRI (3.0 T) system with a dedicated shuttle

**DOI:** 10.1186/s41824-018-0027-1

**Published:** 2018-05-07

**Authors:** Omar Alonso, Gerardo dos Santos, Margarita García Fontes, Henia Balter, Henry Engler

**Affiliations:** 1 Uruguayan Centre of Molecular Imaging (CUDIM), Av. Ricaldoni 2010, 11600 Montevideo, Uruguay; 2 Nuclear Medicine and Molecular Imaging Centre, Hospital de Clínicas, Universidad de la República, Av. Italia S/N, 11600 Montevideo, Uruguay

**Keywords:** Prostate cancer, Biochemical recurrence, ^11^C-Choline, ^68^Ga-PSMA, PET/CT, PET/MRI

## Abstract

**Background:**

The aim of this study was to prospectively compare the detection rate of ^68^Ga-PSMA versus ^11^C-Choline in men with prostate cancer with biochemical recurrence and to demonstrate the added value of a tri-modality PET/CT-MRI system.

**Methods:**

We analysed 36 patients who underwent both ^11^C-Choline PET/CT and ^68^Ga-PSMA PET/CT scanning within a time window of 1-2 weeks. Additionally, for the ^68^Ga-PSMA scan, we used a PET/CT-MRI (3.0 T) system with a dedicated shuttle, acquiring MRI images of the pelvis.

**Results:**

Both scans were positive in 18 patients (50%) and negative in 8 patients (22%). Nine patients were positive with ^68^Ga-PSMA alone (25%) and one with ^11^C-Choline only (3%). The median detected lesion per patient was 2 for ^68^Ga-PSMA (range 0-93) and 1 for ^11^C-Choline (range 0-57). Tumour to background ratios in all concordant lesions (*n* = 96) were higher for ^68^Ga-PSMA than for ^11^C-Choline (110.3 ± 107.8 and 27.5 ± 17.1, mean ± S.D., for each tracer, respectively *P* = 0.0001). The number of detected lesions per patient was higher for ^11^C-Choline in those with PSA ≥ 3.3 ng/mL, while the number of detected lesions was independent of PSA levels for ^68^Ga-PSMA using the same PSA cut-off value. Metastatic pelvic lesions were found in 25 patients (69%) with ^68^Ga-PSMA PET/CT, in 18 (50%) with ^11^C-Choline PET/CT and in 21 (58%) with MRI (3.0 T). MRI was very useful in detecting recurrence in cases classified as indeterminate by means of PET/CT alone at prostate bed.

**Conclusions:**

In patients with prostate cancer with biochemical recurrence ^68^Ga-PSMA detected more lesions per patient than ^11^C-Choline, regardless of PSA levels. PET/CT-MRI (3.0 T) system is a feasible imaging modality that potentially adds useful relevant information with increased accuracy of diagnosis.

## Background

Prostate cancer (PCa) is the second most common malignant tumour in man and one of the most common malignancies in the world (Jemal et al., 2011). Despite the good prognosis the mortality is relatively high especially in more aggressive tumours. Besides, in the period 1990-2010 there was an absolute increase of 100.000 deaths registered worldwide (Lozano et al., 2012).

One of the key clinical issues of this disease is the detection of recurrent disease. To date this is a major challenge for all conventional imaging modalities (Kosuri et al., 2012). PET/CT with radioactively labelled Choline derivatives has found widespread use for the diagnosis of PCa recurrence in patients with biochemical failure.

However, numerous studies have been reported with a relatively low sensitivity and specificity, especially at low prostate specific antigen (PSA) levels (Schmid et al., 2005; Igerc et al., 2008; Kwee & DeGrado, 2008; Hacker et al., 2006; Husarik et al., 2008; Cimitan et al., 2006; Pelosi et al., 2008; Beauregard et al., 2010; Heinisch et al., 2006; Steiner et al., 2009). New tracers with better diagnostic accuracy are needed.

The ^68^Ga-labelled HBED-CC conjugate of the prostate-specific membrane antigen (PSMA) specific pharmacophore based on urea-like structures Glu-NH-CO-NH-Lys (Ga-68 PSMA-HBED-CC) constitutes an attractive and alternative PET tracer for the evaluation of patients with PCa (Perera et al., 2016; von Eyben et al., 2016; Hillier et al., 2009; Eder et al., 2012; Schäfer et al., 2012; Bander, 2006; Liu et al., 1997; Sweat et al., 1998; Mannweiler et al., 2009; Eder et al., 2013; Afshar-Oromieh et al., 2013). Some studies have demonstrated the diagnostic superiority of ^68^Ga-PSMA PET/CT in patients with PCa at biochemical relapse, making intra-individual comparisons with the previous gold standard: radiolabelled Choline (Afshar-Oromieh et al., 2012; Afshar-Oromieh et al., 2014; Schwenck et al., 2017). In this sense, a greater detection rate has been observed, especially evident in the group of patients with low levels of PSA (< 0.5 ng/mL) with the potential of changing clinical management (Schwenck et al., 2017).

Although there are studies that have compared these two radiotracers (with better results for PSMA), only one of them had a prospective design (Morigi et al., 2015).

In addition, the contribution of magnetic resonance imaging (MRI) in hybrid environments (PET/MRI and PET/CT-MRI) has the potential to increase the diagnostic accuracy of ^68^Ga-PSMA scanning as reported by Afshar-Oriomieh et al. (Afshar-Oromieh et al., 2014) and by our group (Alonso et al., 2016) in a preliminary analysis performed in 24 patients that are also included in this study.

Thus, as improved molecular imaging of PCa is necessary, the aim of this study was to prospectively compare the detection rate of ^68^Ga-PSMA versus ^11^C-Choline in men with PCa with biochemical recurrence using a tri-modality PET/CT-MRI system.

## Material and methods

### Patient characteristics

We prospectively analysed 36 patients with biochemical recurrence (defined as PSA > 0.2 ng/mL, PSA doubling time less than 6 months or PSA increase above 2 ng/ml per year), referred to our department between August 2015 and March 2016. Patient characteristics are summarized in Table 1. Twenty-four patients (67%) had undergone radical prostatectomy and 12 patients were treated with radiotherapy (associated or not with androgen deprivation therapy). The average age was 64.7 ± 7.4 years. The median PSA level was 3.3 ng/mL, ranging from 0.2 to 138 ng/mL.

**Table 1:** Patient characteristics and scan results

Patient Number	Age (years)	Treatment	PSA (ng/mL)	^11^C-Choline	^68^G**a**-PSMA	MR (pelvic)
1	67	RT/HT	8.7	Positive	Positive	Positive
2	61	RT/HT	10	Positive	Positive	Positive
3	55	RT/HT	93.85	Positive	Positive	Negative
4	60	Surgery	0.98	Negative	Positive	Negative
5	68	RT/HT	2.33	Positive*	Positive	Positive
6	76	Surgery	0.32	Positive	Positive	Positive
7	58	Surgery	1.32	Negative	Negative	Negative
8	58	Surgery	4.44	Positive	Positive	Positive
9	50	Surgery/RT/QT/HT	3.54	Positive	Positive	Positive
10	63	Surgery	1.7	Positive	Positive	Negative
11	76	RT/HT	138	Positive*	Positive	Positive
12	77	Surgery/HT	6	Positive	Positive	Positive
13	72	Surgery	0.55	Negative	Negative	Negative
14	64	RT/HT	4.33	Equivocal	Positive	Positive
15	71	Surgery/RT/QT/HT	6.5	Positive	Positive	Negative
16	67	Surgery/RT	3,5	Negative	Positive	Negative
17	55	RT	11	Positive	Positive	Positive
18	62	RT/HT	0.4	Positive	Positive	Positive
19	56	Surgery	0.88	Negative	Negative	Negative
20	66	Surgery	1.32	Negative	Negative	Negative
21	63	Surgery	0.3	Negative	Negative	Negative
22	65	Surgery/RT	1.29	Negative	Positive	Negative
23	63	RT	3.96	Positive	Positive	Positive
24	74	RT	7	Positive	Positive	Positive
25	73	Surgery/RT/HT	4.79	Positive	Positive	Positive
26	74	Surgery/RT	0.328	Negative	Negative	Negative
27	69	Surgery/RT/HT	5.8	Equivocal	Positive*	Positive
28	63	RT/HT	2.87	Positive	Equivocal	Positive
29	62	Surgery/HT	2.56	Negative	Positive	Negative
30	72	RT	1.76	Equivocal	Positive	Positive
31	60	Surgery	8.4	Equivocal	Positive	Positive
32	70	Surgery/RT	19	Positive	Positive	Negative
33	45	Surgery	0.2	Negative	Negative	Negative
34	60	Surgery/RT/HT	15	Positive	Positive	Positive
35	72	Surgery	3.16	Equivocal	Positive	Positive
36	63	Surgery	0.2	Equivocal	Equivocal	Positive

RT = Radiation Therapy; HT = Hormonal Therapy; QT = Chemo therapy; MR = Magnetic Resonance; “Positive*” = means “positive” at lymph nodes or bone lesions and “equivocal” at prostate bed

### Compliance with ethical standards

The study was approved by the Ethics Committee of the Uruguayan Centre of Molecular Imaging (CUDIM) and conducted according to the Helsinki Declaration and its subsequent amendments. Additionally, consent to publish and Informed Consents were obtained from all individual participants included in the study.

### Radiopharmaceuticals

#### ^11^C-Choline

Automatized synthesis of [^11^C] N-methyl Choline was performed from ^11^CO_2_ and di-methyl-amino-ethanol (DMAE). Reaction and purification was done on a Sep-Pak Classic CM cartridge (Millipore). Radiochemical purity of ^11^C-Choline was higher than 95%.

#### ^68^Ga-PSMA

^68^Ga-PSMA was produced using PSMA-11 (HBED-CC) from ABX as precursor and ^68^Ga eluted from an^68^Ge/^68^Ga generator (ITG, Germany). The precursor (3,2 to 3,6 nmol) was dissolved in ultrapure water and mixed with 1,00 mL 0,25 M sodium acetate and 650 – 1450 MBq ^68^GaCl_3_ in 4 mL HCl 0.05 M. After 5 min of incubation at 100 °C, ^68^Ga-PSMA was purified by solid phase extraction (Sep-Pak C18 light), formulated with saline and sterilized by filtration. Radiochemical purity was 99.2 ± 1.7%, ^68^Ge breakthrough was less than 2 × 10^− 4^% and of specific activity was 170 ± 76 MBq/nmol.

### Imaging

Patients did not need to fast and were allowed to take all their medications. Patient preparation included the placement of an i.v. line and hydration with at least 500 mL. Within 1-2 weeks all patients underwent a PET/CT scan with ^11^C-Choline and with ^68^Ga-PSMA, randomly performed. PET/CT studies were performed with a 64-slice PET/CT (General Electric Discovery 690 VCT, 64 slices, Waukesha, WI, USA) immediately after the injection of 6.0 MBq/kg ^11^C-Choline or 60 min after the administration of 2.0 MBq/kg ^68^Ga-PSMA. The images were acquired from skull to mid-thigh. The acquisition and processing parameters of PET images were the same for ^11^C-Choline and ^68^Ga-PSMA. CT parameters were as follows: tube voltage 120 kVp, autoMA 80-180 mA, index noise 30, “GE SmartMa dose modulation”, rotation time 0.8 s, rotation length - full helical thickness: 3.75 mm, Pitch 1.375:1 and speed 55 (mm/rot). PET data were acquired in 3D with time-of-flight correction with a scan duration of 3 or 4 min per bed position (^11^C-Choline and ^68^Ga-PSMA, respectively) and with 11-slice overlap. Images were reconstructed using an ordered subset expectation maximization algorithm (OSEM) with time-of-flight correction (matrix size 128 × 128 pixels) with 2 iterations/24 subsets.

For the ^68^Ga-PSMA studies we used a dedicated shuttle for MRI pelvic co-registration: PET/CT-MRI (Discovery 750w 3.0 T, GE Healthcare, Waukesha, WI, USA). An MRI abbreviated protocol was planned with axial panoramic slices (T1 and T2 sequences) starting at the aortic bifurcation. High-resolution thin slices with T2 sequence of the prostate bed as well as focal and panoramic diffusion (DWI) and ADC maps were obtained. MRI images were performed during 30 min, starting 30 min after tracer injection. No contrast media was used for MR or CT scans. Only MRI images of the pelvis were acquired in this study. Whole body MRI was not performed.

Images were evaluated by two board-certified specialists in nuclear medicine and by two board-certified radiologists.

Disagreements were resolved by consensus. ^11^C-Choline and ^68^Ga-PSMA PET/CT scans were randomly evaluated. Lesions that were visually considered suggestive of PCa were counted and analysed regarding their localization (local relapse, bone, lymph node or soft tissue metastases) and their maximum standardized uptake values (SUVmax). We measured the lesion to background ratio (lesion SUVmax/background SUVmax) in all coincident lesions to evaluate the impact on image interpretation. Gluteal musculature was selected as background.

For PET scans, suspicious lesions were defined as any focal uptake, at one or more locations, higher than the nearby background or compared with normal tissue, excluding joint processes and areas of physiological uptake. Lesions were defined as equivocal or indeterminate if the uptake was no typical for malignancy but nevertheless remains unclear or when the urinary physiological activity prevents a correct assessment.

Regarding MR evaluation local recurrence at the prostate gland was described in two clinical scenarios. In the case of patients treated with radiotherapy of the prostate gland, areas of restricted diffusion and low signal were considered as abnormal. In the case of those who underwent prostatectomy, a pathologic image with signal intensity similar to solid tissue and with restricted diffusion was interpreted as local recurrence. Bone metastases were described when there was a low intensity lesion in T1 and T2, or show restricted diffusion. Lymph nodes were considered positive if their size was > 12 mm.

This paper analyzes only detection rates of the radiopharmaceuticals. Validation of findings using histology and/or clinical follow-up is needed in order to calculate accuracy diagnostic values.

Image analysis was performed using a General Electric AW 4.6 platform (HP Workstation and LINUX OS) and OsiriX v6.5.2 (OS X El Capitan).

### Statistical analysis

Tumour to background ratio signal from the same lesions in both ^68^Ga-PSMA and ^11^C-Choline studies were analysed using the Student paired t-test. Comparisons of the number of detected lesions per patient in each study, and with each radiotracer regarding PSA levels, were done with the two-sided Mann–Whitney test. Additionally, the number of pelvic lesions detected with Choline, PSMA and MRI were compared using the Kruskal-Wallis (ANOVA) and Fisher tests. A *P* value < 0.05 was considered significant. For statistical analyses, we used GraphPad Prism version 7.00 for Mac OS X, La Jolla, CA, USA.

## Results

**Fig. 1:**
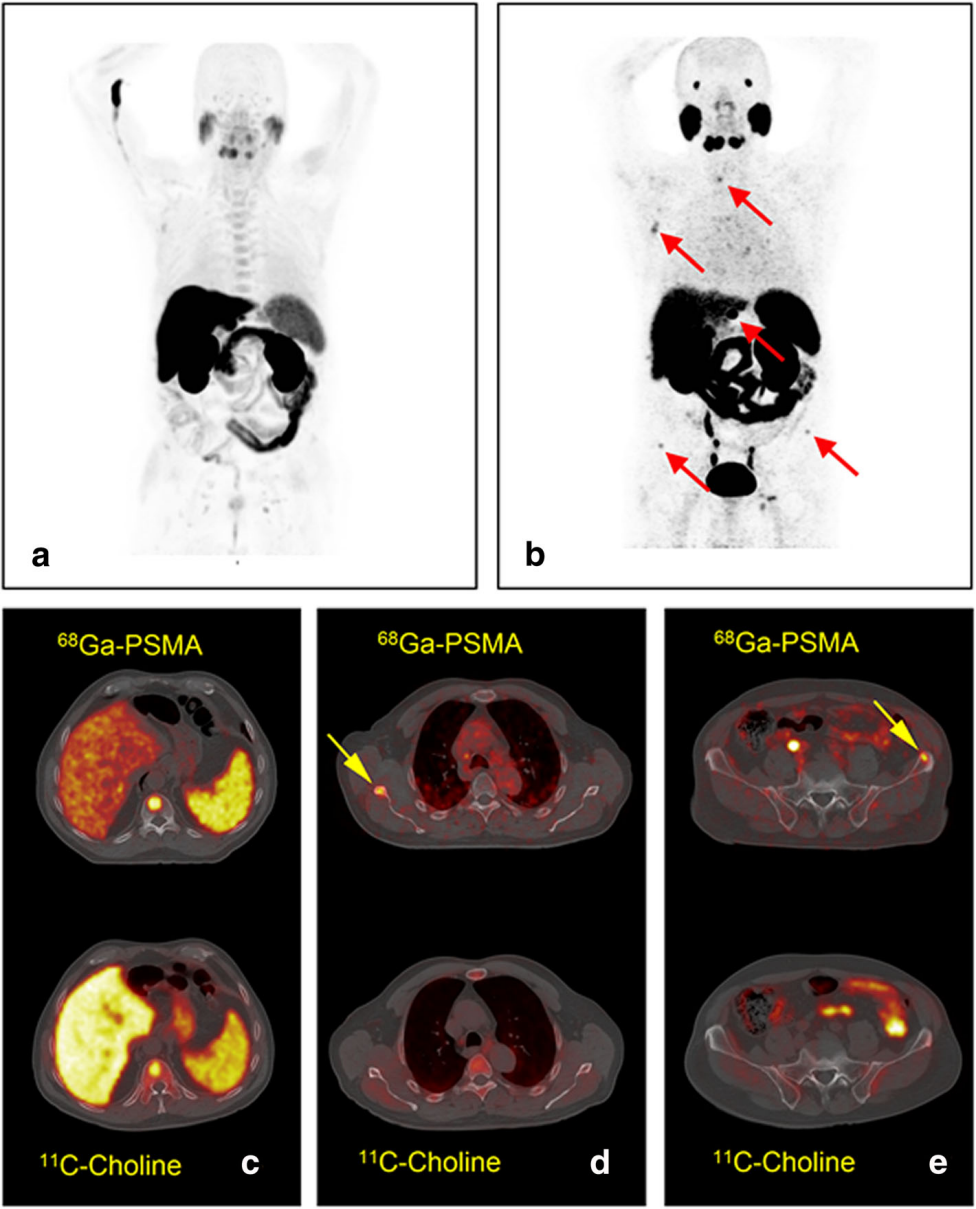
Patient 1. A 67-year-old patient treated with radiotherapy and hormonotherapy with PSA relapse (PSA level 8.7 ng/ml). MIP images with bone metastases (red arrows), scanned with ^11^C-Choline (**a**) and with ^68^Ga-PSMA (**b**). Metastasis in T11 demonstrated with both tracers (**c**). Right scapula (**d**) and left iliac crest (**e**) focal lesions demonstrated only with ^68^Ga-PSMA (yellow arrows)

**Fig. 2:**
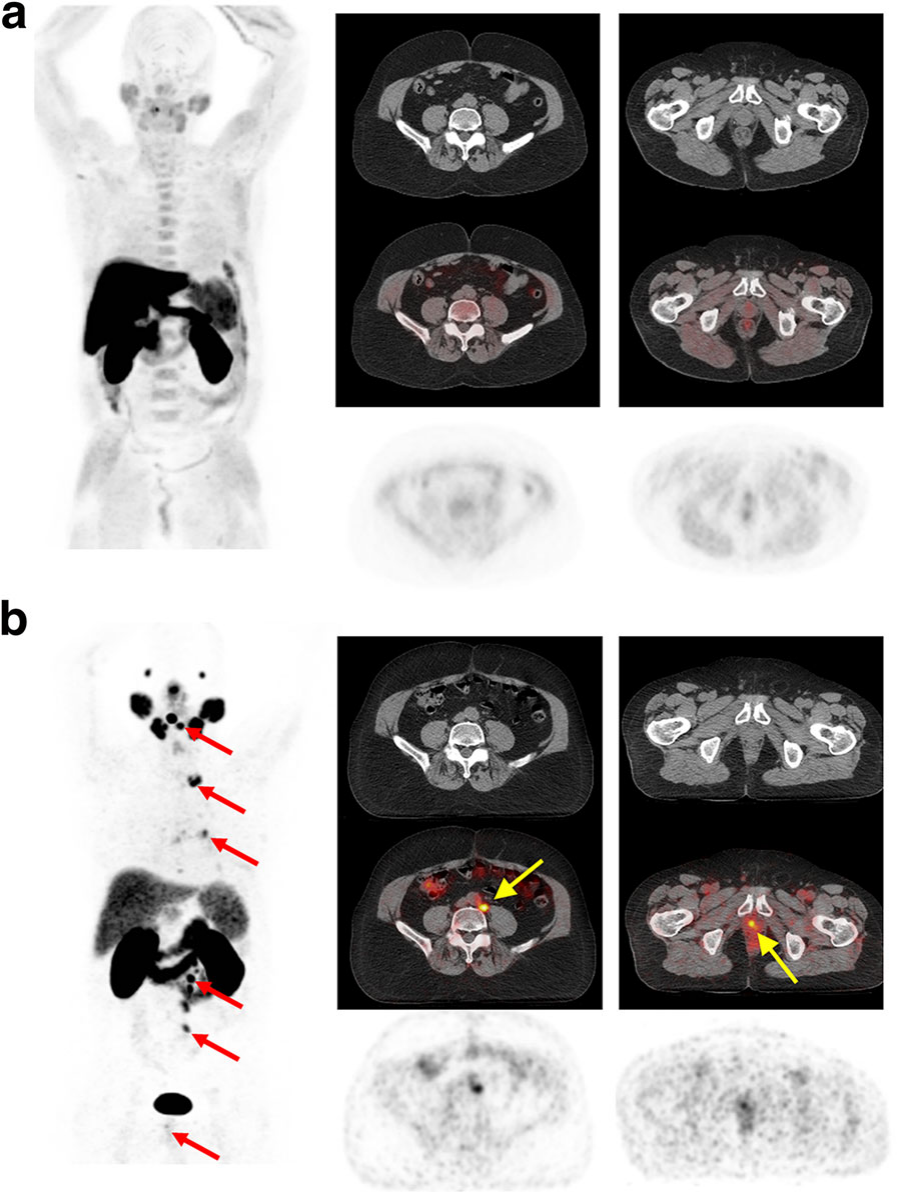
Patient 5. A 68-year-old patient with PCa treated with radiotherapy and complete androgen blockade with current elements of biochemical relapse (PSA level 2.33 ng/ml), scanned with ^11^C-Choline (**a**) and with ^68^Ga-PSMA (**b**).Several metastases are demonstrated only by means of ^68^Ga-PSMA alone (red arrows). Yellow arrows show a metastatic retroperitoneal lymph node and a local relapse in ^68^Ga-PSMA PET/CT, while ^11^C-Choline PET/CT showed no suspicious lesion in these areas

Overall detection rate was 75% (27/36) for ^68^Ga-PSMA and 53% (19/36) for ^11^C-Choline. Both scans were positive in 18 patients (50%) and negative in 8 patients (22%). Nine patients were positive with ^68^Ga-PSMA alone (25%) and one with ^11^C-Choline only (3%), (Figs. 1 and 2).

A total of 185 lesions were detected by at least one radiopharmaceutical: 183 for ^68^Ga-PSMA and 98 for ^11^C-Choline. Lesion localization for each tracer is described in Table 2. The median detected lesion per patient was 2 for ^68^Ga-PSMA (range 0-93) and 1 for ^11^C-Choline (range 0-57). The difference was statistically significant (*P* = 0.023).

**Table 2:** Lesion localization according to technique

	^11^C-Choline					^68^Ga-PSMA					Pelvic MR				
Patient No	Prostate bed	Lymph nodes	Bone	Soft tissue	Total	Prostate bed	Lymph nodes	Bone	Soft tissue	Total	Prostate bed	Lymph nodes	Bone	Soft tissue	Total
1	1	0	3	0	4	1	4	5	0	10	1	0	0	0	1
2	1	0	0	0	1	1	0	0	0	1	1	0	0	0	1
3	1	18	38	0	57	1	27	65	0	93	0	0	0	0	0
4	0	0	0	0	0	0	2	0	0	2	0	0	0	0	0
5	0	2	1	0	3	1	10	1	0	12	2	0	0	0	2
6	1	0	0	0	1	1	0	0	0	1	1	0	0	0	1
7	0	0	0	0	0	0	0	0	0	0	0	0	0	0	0
8	0	4	0	0	4	0	3	0	0	3	0	4	0	0	4
9	0	2	2	0	4	0	2	2	0	4	0	2	2	0	4
10	0	1	0	0	1	0	1	1	0	2	0	0	0	0	0
11	0	0	1	0	1	1	0	4	0	5	1	0	0	0	1
12	1	0	0	0	1	1	0	0	0	1	1	0	0	0	1
13	0	0	0	0	0	0	0	0	0	0	0	0	0	0	0
14	0	0	0	0	0	1	5	1	0	7	2	0	0	0	2
15	0	5	0	0	5	0	12	0	0	12	0	0	0	0	0
16	0	0	0	0	0	0	1	0	0	1	0	0	0	0	0
17	2	0	0	0	2	2	0	0	0	2	2	0	0	0	2
18	1	0	0	0	1	1	0	0	0	1	1	0	0	0	1
19	0	0	0	0	0	0	0	0	0	0	0	0	0	0	0
20	0	0	0	0	0	0	0	0	0	0	0	0	0	0	0
21	0	0	0	0	0	0	0	0	0	0	0	0	0	0	0
22	0	0	0	0	0	0	1	0	0	1	0	0	0	0	0
23	1	0	0	0	1	1	0	0	0	1	1	0	0	0	1
24	1	2	0	0	3	1	2	0	0	3	1	0	0	0	1
25	0	2	2	0	4	0	2	2	0	4	0	0	1	0	1
26	0	0	0	0	0	0	0	0	0	0	0	0	0	0	0
27	0	0	0	0	0	0	7	0	0	7	1	0	0	0	1
28	1	0	0	0	1	0	0	0	0	0	1	0	0	0	1
29	0	0	0	0	0	0	3	0	0	3	0	0	0	0	0
30	0	0	0	0	0	1	0	0	0	1	1	0	0	0	1
31	0	0	0	0	0	1	0	0	0	1	1	0	0	0	1
32	1	0	1	0	2	1	0	1	0	2	0	0	0	0	0
33	0	0	0	0	0	0	0	0	0	0	0	0	0	0	0
34	0	2	0	0	2	0	2	0	0	2	0	2	0	0	2
35	0	0	0	0	0	1	0	0	0	1	1	0	0	0	1
36	0	0	0	0	0	0	0	0	0	0	1	0	0	0	1
Total	12	38	48	0	98	17	84	82	0	183	20	8	3	0	31

Tumour to background ratios in all concordant lesions (*n* = 96) were higher for ^68^Ga-PSMA than for ^11^C-Choline (110.3 ± 107.8 and 27.5 ± 17.1, mean ± S.D., for each tracer, respectively *P* = 0.0001).

When compared the detection rate for each tracer according to PSA levels, we found that the number of detected lesions per patient was higher for ^11^C-Choline in those with PSA ≥ 3.3 ng/mL(median value of our patient sample): 0.5 (0-3) versus 2 (0 -57), median (range), respectively, (*P* = 0.03). On the other hand, the number of detected lesions was independent of PSA levels for ^68^Ga-PSMA using the same PSA cut-off value: 1 lesion per patient if PSA ≥ 3.3 (range 0 – 12) versus 3 lesion per patient if PSA < 3,3 (range 1-93),(*P* = 0.05).

**Fig. 3:**
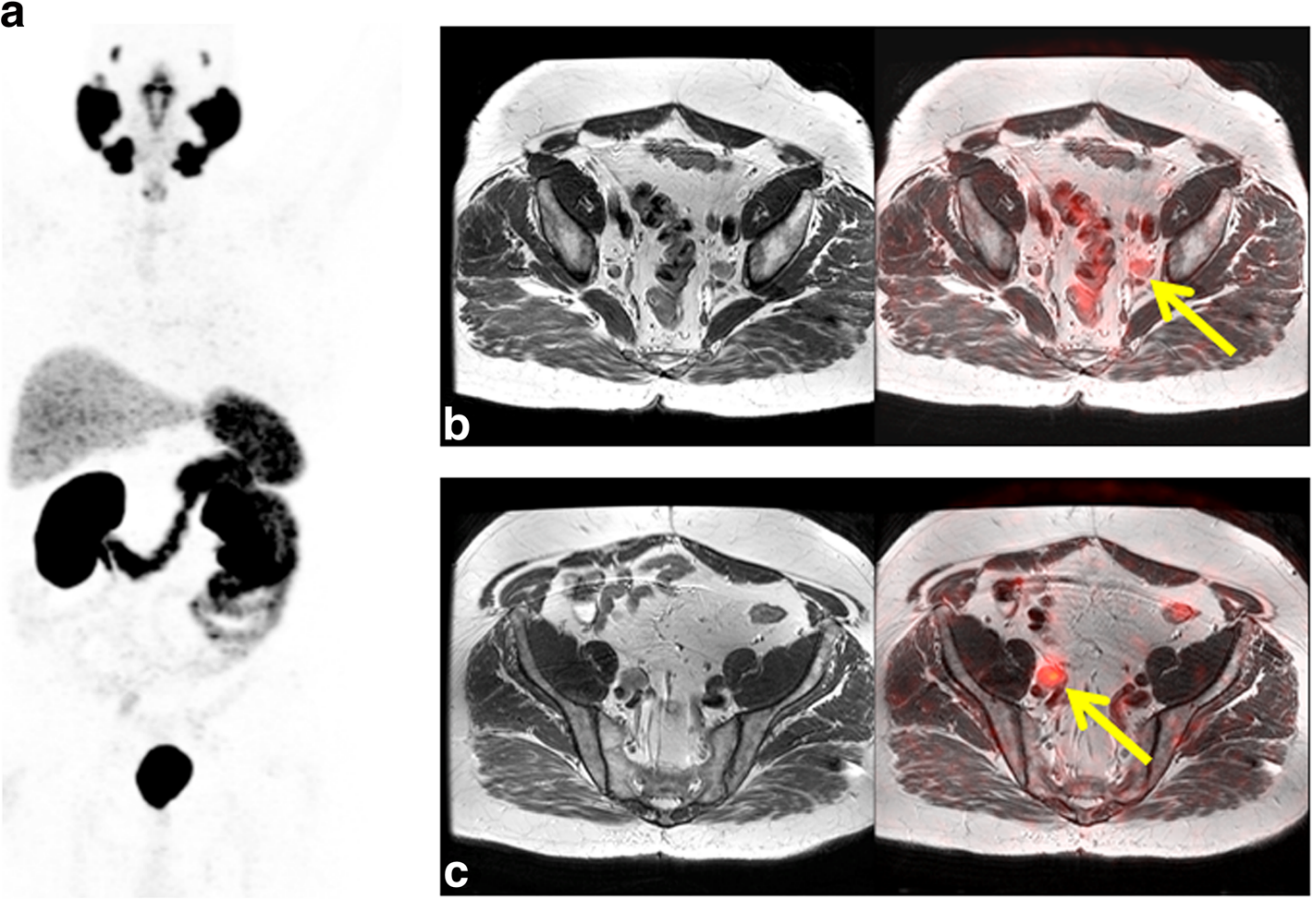
Patient 8. A 58-year-old-patient with PSA relapse (PSA level 4.44 ng/ml) after radical prostatectomy. MIP (**a**), MR and ^68^Ga-PSMA PET/MR (**b**-**c**) images showing pelvic lymph node metastasis (yellow arrows)

**Fig. 4:**
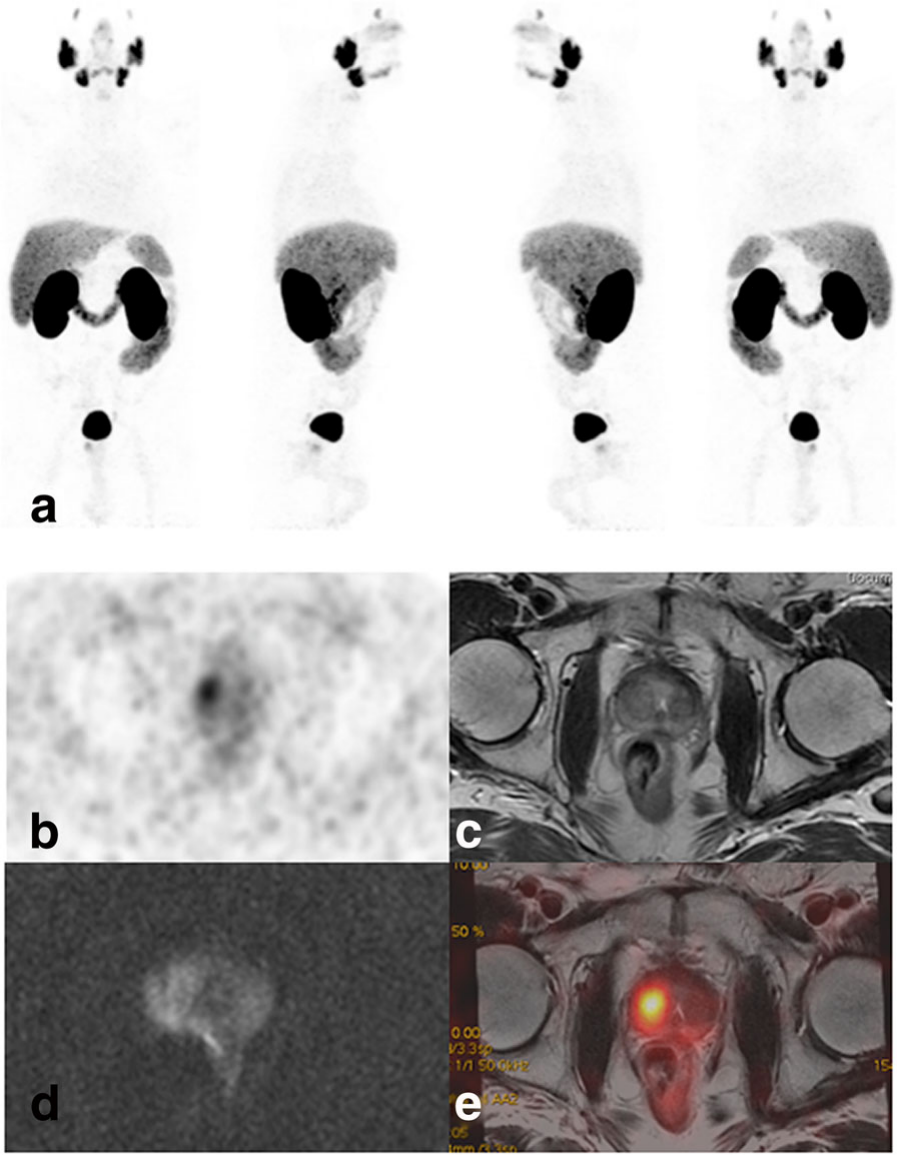
Patient 2. A 61-year-old-patient treated with radiotherapy and hormonotherapy with PSA relapse (PSA level 10 ng/ml). MIP and axial ^68^Ga-PSMA PET (**a**-**b**), high-resolution T2 (**c**), DWI (**d**) and PET/MR images (**e**) demonstrating a coincident local relapse. DWI shows peripheral diffusion restriction that partially overlaps the lesion seen in T2 and perfectly matches PSMA uptake

**Fig. 5:**
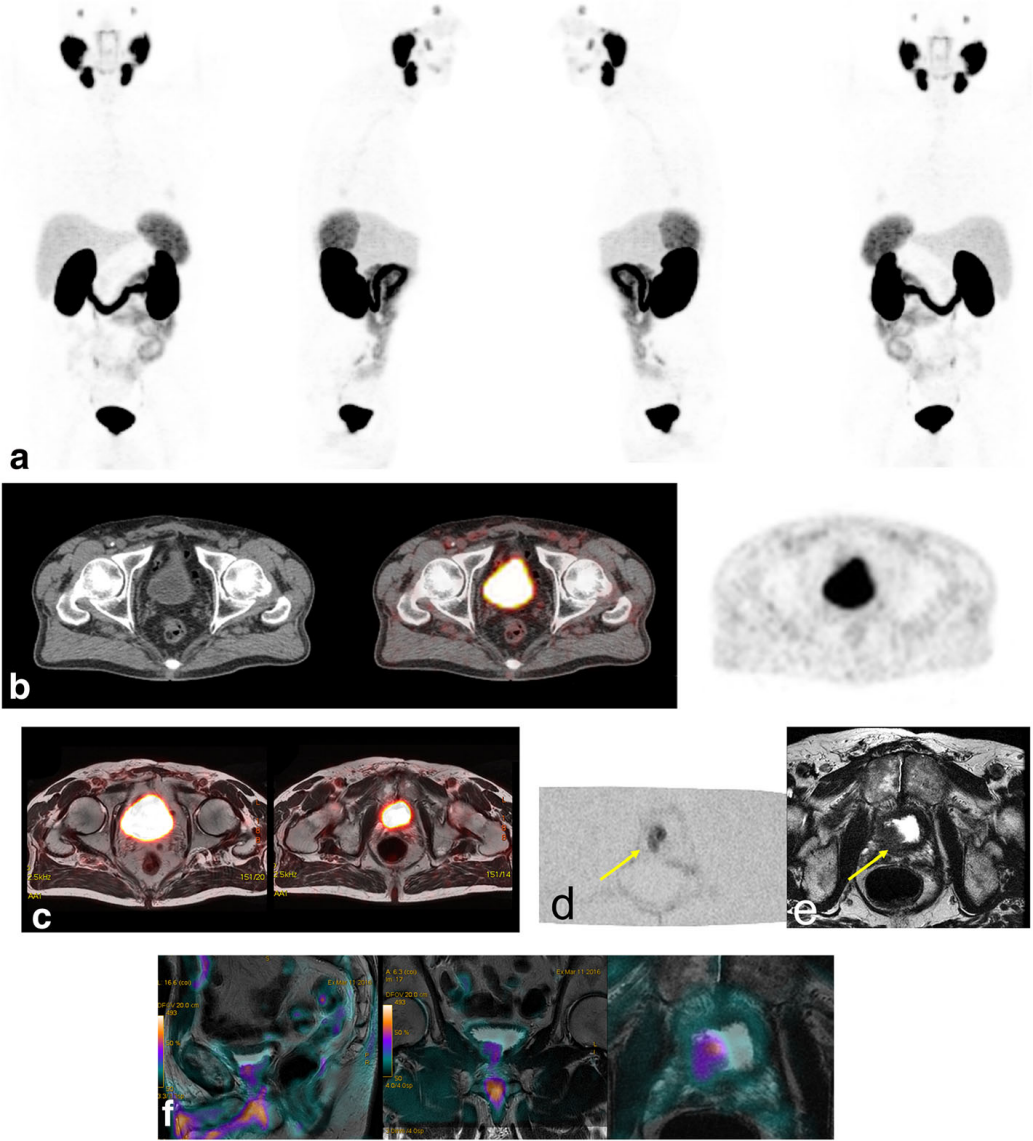
Patient 36. MIP ^68^Ga-PSMA images (**a**) of a 68-year-old patient with PCa treated with surgery with current elements of biochemical relapse (PSA level 0.2 ng/ml).^68^Ga-PSMA PET/CT (**b**) and ^68^Ga-PSMA PET/MR images (**c**) without abnormal pelvic findings. DWI (**d**) and high-resolution T2 (**e**) MR scans show a local relapse lesion with restriction (arrows) that was hidden by physiological tracer bladder activity. The hypointense lesion involves the infundibulum in the right posterolateral prostate region. MR-DWI fusion images are shown in (**f**)

Concerning pelvic evaluation, metastatic lesions were found in 25 patients (69%) with^68^Ga-PSMA, 18 (50%) with ^11^C-Choline and 21 (58%) with MRI (3.0 T). Pelvic MRI detected 31 lesions: 20 at the prostate bed (local recurrence), 8 lymph nodes and 3 bone lesions, as shown in Table 2. Figures 3 and 4 show ^68^Ga-PSMA MIP and PET/MRI images demonstrating coincident metastatic pelvic lymph nodes and a local relapse, respectively (^11^C-Choline images not shown). Besides, MRI was very useful in detecting recurrence in cases classified as indeterminate by means of PET/CT alone at prostate bed as shown in Fig. 5. Nine patients in total were classified as indeterminate or equivocal (patients 5, 11, 14, 27, 28, 30, 31, 35 and 36), with at least one of the tracers: six patients with ^11^C-Choline (patients 5, 11, 14, 30, 31, and 35), one patient with ^68^Ga-PSMA (patient 28) and two patients with both tracers were indeterminate, (patients 27 and 36) as shown in Table 2. When PET/CT was unable to detect a local relapse due to physiological tracer activity in the bladder, the MRI component was clearly pathological.

Of these nine patients, four had bone or lymph node metastasis detected with at least one of the tracers as shown in Table 2 (patients 5, 11, 14 and 27), rendering the added value of MRI in those cases less important. It is well known that having a local recurrence in the prostatic bed is of minor importance if metastases are present at the same time.

No indeterminate or equivocal results were found at lymph nodes or bone lesions.

## Discussion

After applying curative intent treatment for clinically localized PCa with surgery or radiotherapy, 15-30% of patients show biochemical progression (Cher et al., 1998), which precedes a clinically detectable recurrence at the pelvis or metastatic disease within a period of months or years (Moul et al., 2000; Roberts et al., 2001). Furthermore, the detection of lesions associated with a recurrence of PCa in the context of a biochemical relapse constitutes a major challenge for all imaging modalities including Choline PET/CT (Schmid et al., 2005; Igerc et al., 2008; Kwee & DeGrado, 2008; Hacker et al., 2006; Husarik et al., 2008; Cimitan et al., 2006; Pelosi et al., 2008; Beauregard et al., 2010; Heinisch et al., 2006; Steiner et al., 2009).

The usefulness of PET/CT with radiolabelled Choline in the assessment of these patients has been amply demonstrated in several clinical studies (Perera et al., 2016; von Eyben et al., 2016; de Jong et al., 2003; Picchio et al., 2003; Rinnab et al., 2007), which have confirmed a preferential uptake by PCa, lymph nodes and their metastases (Hara et al., 1998; Kwee et al., 2005; Yamaguchi et al., 2005). However, Choline is not specific for prostate cancer either in intra prostatic or extra prostatic disease and a high affinity of the radiopharmaceutical has been evidenced by benign hyperplasia. As a consequence, discrimination between benign and malignant intraprostatic tissue is hampered by low specificity (Souvatzoglou et al., 2011; Farsad et al., 2005). Moreover, the sensitivity of Choline PET/CT in detecting locally recurrent PCa is low, notably in the case of low PSA values (Krause et al., 2008; Giovacchini et al., 2013; Castellucci et al., 2009; Castellucci et al., 2011; Mamede et al., 2013).

PSMA is a transmembrane protein with a significantly increased expression in PCa cells (Silver et al., 1997), recently selected as a target for molecular imaging approaches (Mease et al., 2013). Several clinical studies showed additional advantages of ^68^Ga-PSMA PET/CT in comparison with radioactively labelled analogues of Choline (Afshar-Oromieh et al., 2014; Eiber et al., 2015), a high image contrast due to low background signal, sensitive detection of small lesions due to high radiotracer uptake, and improved detection rates of recurrent prostate cancer and metastases especially at low PSA levels. However, labelled PSMA is also not specific for prostate cancer and there is increasing evidence suggesting that PSMA can be expressed in other solid tumours. This fact can limit the technique’s specificity (Silver et al., 1997).

We prospectively analysed 36 patients who underwent both ^11^C-Choline PET/CT and ^68^Ga-PSMA PET/CT analysis within a time window of 1-2 weeks. Additionally, for the ^68^Ga-PSMA scan, we used a PET/CT-MRI (3.0 T) system with a dedicated shuttle, acquiring MRI images of the pelvis. In all cases, there was biochemical recurrence following prior conventional treatment of PCa.

Although there is controversy about the influence of androgen deprivation therapy (ADT) in the detection rate of PET/CT with ^68^Ga-PSMA, it has been shown that the probability of a pathological ^68^Ga-PSMA-11 PET/CT scan is strongly associated with ongoing ADT (Afshar-Oromieh et al., 2017).

The use of ADT in our patient sample was not an exclusion criteria and its impact on the detection rate remains unknown.

In 75% of the patients at least one suspicious lesion for PCa was detected with ^68^Ga-PSMA PET/CT whereas only 53% of the patients presented pathological findings with ^11^C-Choline PET/CT. Our results were like those reported in the literature (Schmid et al., 2005; Igerc et al., 2008; Kwee & DeGrado, 2008; Hacker et al., 2006; Husarik et al., 2008; Cimitan et al., 2006; Pelosi et al., 2008; Beauregard et al., 2010; Heinisch et al., 2006; Steiner et al., 2009; Afshar-Oromieh et al., 2013). ^11^C-Choline PET/CT did not reveal any suspicious lesions in 17 patients, while only 9 patients presented without any pathological findings in ^68^Ga-PSMA PET/CT.

^68^Ga-PSMA PET/CT detected significantly more PCa lesions when compared to Choline. These data are also similar to our previous report concerning this PSMA ligand (Afshar-Oromieh et al., 2013). In addition, the detection rates of Choline agree with the data reported in the literature (Schmid et al., 2005; Igerc et al., 2008; Kwee & DeGrado, 2008; Hacker et al., 2006; Husarik et al., 2008; Cimitan et al., 2006; Pelosi et al., 2008; Beauregard et al., 2010; Heinisch et al., 2006; Steiner et al., 2009). We also demonstrated a significantly higher tumour to background ratio with ^68^Ga-PSMA than with ^11^C-Choline. Similar results have been reported in the literature (Afshar-Oromieh et al., 2014; Morigi et al., 2015).Therefore, ^68^Ga-PSMA PET/CT proved to be clearly superior in detecting PCa lesions compared to Choline-based PET/CT, especially at low PSA levels.

Even though the literature sets the positivity of ^68^Ga-PSMA PET/CT dependent on the PSA-value (PSA < 0.5), in this study only 6 patients have PSA-values < 0.5 ng/mL, and 27 patients have PSA > 1 ng/mL (Table 1).

As a result, the statistical cut-off point criteria was set according to the PSA values of the sample i.e. (median PSA value = 3.3 ng/mL). Unfortunately, our study did not include enough patients with lower PSA values who can potentially benefit from salvage treatment.

Despite the small number of patient enrolled, this study reinforces the high detection rate for ^68^Ga-PSMA at low PSA levels, compared to ^11^C-Choline in a prospective trial design.

In the last few years, the advent of new molecular imaging methods, such as MRI may provide clinicians with useful information that can have an impact on the management of PCa patients. Multiparametric MRI has shown high sensitivity and specificity for the detection of local and regional recurrence after treatment in PCa patients (de Rooij et al., 2014). PET/MRI with ^68^Ga-PSMA has recently been introduced with promising results. This combination allows excellent morphological detail, multi-parameter functional information and molecular information data. In this context, the tri-modality PET/CT–MRI system includes the transfer of the patient on a dedicated shuttle from one modality to another without changing patient position (Veit-Haibach et al., 2013). This novel sequential imaging technique could lead to a significant improvement in the detection of PCa.

Afshar-Oromiehet al. (Afshar-Oromieh et al., 2014) and Eiber et al. (Eiber et al., 2016) evaluated the feasibility of PET/MRI hybrid system with ^68^Ga-PSMA. In their studies, they demonstrated that prostate cancer was detected more easily and more accurately with the ^68^Ga-PSMA PET/MRI hybrid system, than with PET/CT, and with less radiation exposure. Consequently, this new technique could clarify inconclusive PET/CT results.

Regarding pelvic evaluation, metastatic lesions were found in 25 patients (69%) with ^68^Ga-PSMA, 18 (50%) with ^11^C-Choline and 21 (58%) with MRI (3.0 T). The detection rate of ^68^Ga-PSMA PET/CT was greater than MRI in the detection of pelvic lymph nodes as the literature has described (Gupta et al., 2017; Tulsyan et al., 2017). Advantages of the trimodality PET/CT-MRI system include a more accurate attenuation correction, reliable PET-quantification, superior soft tissue contrast and a higher imaging flexibility that improves diagnostic accuracy for PCa. (de Rooij et al., 2014; Veit-Haibach et al., 2013). The sequential acquisition of the techniques reduces the problems related to the lack of alignment and the change of position of the patient. An important finding in our study is that MRI was highly useful in detecting recurrence in cases classified as indeterminate at prostate bed by means of PET/CT alone, especially in patients with local recurrences detected near the bladder as described in the literature (Afshar-Oromieh et al., 2014; Eiber et al., 2016) and shown in Fig. 5. In those patients PET/CT was unable to detect a local relapse due to physiological tracer activity in the bladder, while MR was clearly pathological. In cases where PET/CT shows no local recurrence and high suspicion of local relapse persists, it would be advisable to complete the study with an MRI of the region, given the greater performance of this method in this scenario.

## Conclusion

In patients with prostate cancer with biochemical recurrence ^68^Ga-PSMA detected more lesions than ^11^C-Choline regardless of PSA levels.PET/CT-MRI (3.0 T) system is a feasible imaging modality that potentially adds useful relevant information with increased accuracy of diagnosis.

## Abbreviations

ADT: androgen deprivation therapy
DMAE: Di-methyl-amino-ethanol
MRI: Magnetic Resonance Imaging
PCa: Prostate Cancer
PSA: Prostate Specific Antigen
PSMA: Prostate-specific Membrane Antigen
